# Editorial: The Complexity of Primary Antibody Deficiencies

**DOI:** 10.3389/fimmu.2021.635482

**Published:** 2021-04-21

**Authors:** Isabella Quinti, Giuseppe Spadaro, Stephen Jolles, Antonio Condino-Neto

**Affiliations:** ^1^Department of Molecular Medicine, Sapienza University of Rome, Rome, Italy; ^2^Department of Translational Medical Sciences and Center for Basic and Clinical Immunology Research (CISI), University of Naples Federico II, Naples, Italy; ^3^Immunodeficiency Centre for Wales, University Hospital of Wales, Cardiff, United Kingdom; ^4^Department of Immunology, Institute of Biomedical Sciences, University of São Paulo, São Paulo, Brazil

**Keywords:** primary antibody deficiencies agammaglobulinemia, common variable immune deficiencies, chronic lung disease, liver diseases, endocrine diseases, T helper follicular cells, free light chains

##  

Primary antibody deficiencies represent by far the largest group of primary immunodeficiencies (PID) at 56% (ESID Registry), however the proportion of patients in whom antibody deficiency represents a component of their condition is greater still at around 75% (1). It has become clear over recent years that while the vast majority of such patients experience recurrent infections a significant proportion are also affected by non-infectious dysregulatory complications which include malignancy, autoimmunity, inflammation and allergy (**Figure 1**). The increasingly complex manifestations of Primary Antibody Deficiencies have a major impact on the clinical management of patients and raise diagnostic and therapeutic challenges (2). This Research Topic draws together a series of reports focusing on a range of these non-infectious complications and their clinical implications.

**Figure 1:**
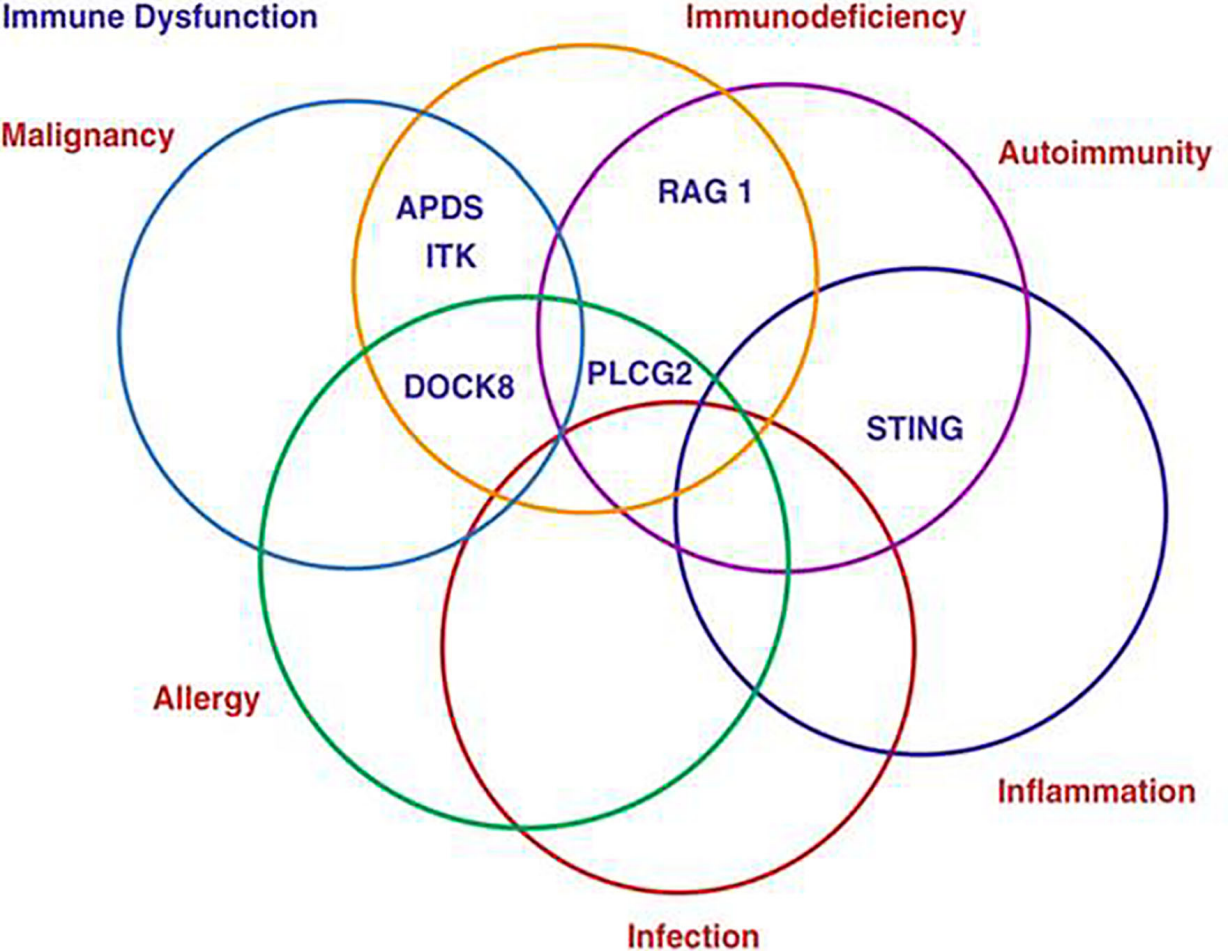
The complexity of Primary Antibody Deficiencies.

## Genetics

In recent years, our understanding of genetics of primary antibody deficiencies (PAD) (1) further progressed unraveling an independent role of minor and major histocompatibility complex genes and common genetic variants as well as monogenic forms as shown by Abolhassani et al. who identified the most significant partial haplotype linked with the unsolved CVID as W*01:01:01-DMA*01:01:01-DMB*01:03:01:02-TAP1*01:01:01. Beside the high number of BTK mutations so far described (2), some atypical clinical manifestations have been described in XLA patients and linked to a novel hemizygous c.1632-1G>A mutation in the *BTK* gene as shown by Han et al. in a child with atypical X-Linked Agammaglobulinemia and recurrent hemophagocytosis (HLH) whose remission of HLH episodes was finally achieved after he received monthly Ig replacement therapy as the only treatment for HLH.

## Patient Management

As outlined, further advances in understanding key aspects of primary antibody deficiency associated conditions should assist in advances in diagnosis and management. New aspects of the clinical complexity of PAD was reported by Coopmans et al. who suggested that an assessment of the endocrine axis should be considered since a high prevalence of both anterior pituitary and end-organ endocrine dysfunction (secondary hypothyroidism, secondary hypogonadism, premature ovarian failure, primary testicular failure, partial adrenal insufficiency, severe growth or mild hormone deficiency) were identified in adult PAD patients causing a considerable health burden. A detailed description of CVID-associated non-infectious complication was reported by Ho and Cunningham-Rundles. Autoimmunity, chronic lung disease, lymphoid hyperplasia/splenomegaly, liver disease, granulomas, gastrointestinal disease, lymphoma and other malignancies were found in two third of CVID patients. These complications may be present in the same patient, and progress to a wide spectrum of associated sequelae. An aggressive multidisciplinary approach may reduce such progression. Further exacerbations, as for permanent lung damage and bronchiectasis, may develop even in the absence of known infections, as shown by Wall et al. Similarly, in the liver, granulomas and nodular regenerative hyperplasia progressing to portal hypertension (3) affected about 50% of CVID patients without or with monogenic forms included in the clinical spectrum of CVID such as ICOS, NFKB1, NFKB2, CTLA-4, PI3Kδ pathway, ADA2, and IL21-R genetic defects, as shown by Antonio Pecoraro et al.

## Pathogenesis

The hallmarks of CVID are hypogammaglobulinemia, low frequency of isotype-switched memory B cells, and compromised B-cell differentiation into memory or antibody-secreting cells (4). Further insight on cellular defects underlying CVID pathogenesis are illustrated by Carsetti et al. who showed that circulating IgM memory B cells have a distinctive role in mucosal protection and suggested the existence of a functional gut-spleen axis where TACI-expressing IgM memory B cells producing IgA were localized under the epithelial cell layer where the TACI ligand APRIL was extremely abundant. The impairment of mucosal immunity might result in less diverse and significantly altered bacterial, but not fungal gut microbiota, in CVID patients, apparently associated with a more severe disease phenotype as shown by Fiedorová et al. Although described as a B cell intrinsic disease, numerous abnormalities have been reported in other immune cell compartments as in follicular helper T cells, a CD4+ T cell population specialized in B cell help as described by Le Saos-Patrinos et al., and by Gereige and Maglione who address the aspects of immune dysregulation associated with autoimmunity, including elevations of T helper type 1 and follicular helper T cells and B cells expressing low levels of CD21 as well as a decrease in regulatory T cells.

## Diagnosis

An often unresolved aspect in the management of antibody deficiencies is the differential diagnosis with secondary forms of hypogammaglobulimias, and in particular those associated with lymphoproliferative diseases (5). Scarpa at al. proposed that serum free light chains analysis might have a role in differential diagnosis of CVID from other causes of hypogammaglobulinemia and in the early detection of monoclonal lymphoproliferation occurring over years. Overall, CVID patients presented a low κ and λ chain concentration. The most common pattern was κ−λ−, followed by κ−λ+, κ+λ+, and κ+λ−, while in secondary forms it was κ+λ+.

## Controversies

Experience of solid organ transplantations, and hematopoietic stem cell transplantation in patients with primary antibody deficiency remains limited and this aspect of management needs further study alongside the developing potential of gene therapy and gene editing.

## Conclusions

Together the articles comprising this Research Topic provide important and timely updates about the current status of non-infectious diseases in primary antibody deficiencies, and in particular in CVID. Each report raises questions and indicates aspects that require further attention and scientific enquiry.

## Author Contributions

All authors listed have made a substantial, direct, and intellectual contribution to the work and approved it for publication.

## Funding

AC-N is supported by a grant from the Jeffrey Modell Foundation and Fundação de Amparo a Pesquisa do Estado de Sao Paulo (FAPESP 2016/22158-3).

## Conflict of Interest

The authors declare that the research was conducted in the absence of any commercial or financial relationships that could be construed as a potential conflict of interest.
